# Different Facets of Creativity in Employees Covering Non-Clinical to Clinical Manifestations of Burnout

**DOI:** 10.3390/jintelligence10040105

**Published:** 2022-11-14

**Authors:** Elisabeth M. Weiss, Markus Canazei, Corinna M. Perchtold-Stefan, Christian Rominger, Ilona Papousek, Andreas Fink

**Affiliations:** 1 Department of Psychology, University of Innsbruck, 6020 Innsbruck, Austria; 2 Department of Psychology, University of Graz, 8010 Graz, Austria

**Keywords:** burnout, creativity, cognition, depression, sleep quality

## Abstract

Empirical studies exploring the relationship between burnout and creativity are very rare. In the present study, a well-defined group of clinical burnout patients (n = 75) and two groups of working people showing high (n = 39) vs. low burnout symptoms (n = 62) were investigated. Participants completed various creativity tests including self-assessed facets of creativity, as well as psychometric measures of figural and verbal creativity. Furthermore, we examined individual and clinical characteristics that may influence creativity in burnout patients, such as depression, sleep quality, daytime fatigue, and cognitive measures (i.e., selective attention and executive control). The clinical burnout group scored lowest in all creativity tasks and cognitive tests. Additionally, they showed lower nighttime sleep quality and higher depression scores. However, creativity scores in both groups of working people were largely comparable, indicating that only more severe (i.e., clinical) manifestations of burnout are linked to creativity.

## 1. Introduction

Creativity is regarded as a key to personal and organizational social success. Creative thinking includes the ability to generate new ideas and approaches to find new solutions (Hennessey and Amabile 2010), along with the ability to meet the challenges of our everyday life in a flexible and appropriate manner (Runco 2004; Sternberg and Lubart 1996). It is commonly assumed that uncontrollable stress and high social-evaluative contexts have a negative effect on creativity (e.g., meta-analysis of Byron et al. 2010), as well as on higher order cognitive capacity, which is related to creativity, including task switching and cognitive flexibility (Steinhauser et al. 2007; Plessow et al. 2011, 2012).

During the last decade, there has been a large amount of research focused on work-related chronic stress and burnout. Probably the most prominent definition of burnout by Maslach and Jackson (1981) describes the three key dimensions of burnout, namely emotional exhaustion (feeling of being emotionally overextended and exhausted with one’s work), depersonalization (development of negative and indifferent attitudes toward others), and reduced personal accomplishment (loss of feelings of self-competence and not being satisfied with one’s achievements; Maslach et al. 2001). It is well-documented that rates of stress-related symptoms and burnout are significantly increased in health care providers (e.g., Shah et al. 2021; Dimitriu et al. 2020), students (e.g., Bauernhofer et al. 2019; Kaggwa et al. 2021), teachers (e.g., Pressley 2021). or expatriates (e.g., Fu and Charoensukmongkol 2021). Previous studies found substantial variability in prevalence rates of burnout ranging from 2.5 to 80.5% (Adriaenssens et al. 2015; Rotenstein et al. 2018). Methodological heterogeneities between studies, such as different definitions of burnout, assessment instruments, or study populations may partly account for these inconsistent findings. Moreover, several studies reported that demographic variables (e.g., age, gender, or profession) (Ahola et al. 2006; Lam et al. 2022; Stenlund et al. 2007), work characteristics and job demands (e.g., role conflicts, work overload, work pressure, or work interruptions) (Lam et al. 2022; Weigl et al. 2012), personality characteristics (e.g., trait anxiety) (Voultsos et al. 2020), neuroticism (Roloff et al. 2022), coping strategies (e.g., mindfulness) (Charoensukmongkol and Puyod 2022), social support, cognitive reappraisal, or problem-focused coping (Shin et al. 2014)) were associated with burnout.

Until now, there has been a lack of a consensual definition of burnout (Demerouti and Bakker 2008; Maslach et al. 2001; Shirom and Melamed 2006), and there is substantial overlap in symptomatology with other psychiatric disorders, such as chronic fatigue (Huibers et al. 2003) and depression (Ahola et al. 2014; Bianchi et al. 2015). Therefore, the diagnosis is only included in the chapter: “Factors influencing health status or contact with health services” (QD86 burnout) in the International Classification of Diseases and Related Health Problems (ICD-11: World Health Organization 2019) and is not classified in the Diagnostic and Statistical Manual of Mental Disorders (DSM-5; American Psychiatric Association 2013).

Empirical studies exploring the relationship between burnout and creativity are rare, primarily focusing on specific occupational groups, such as teachers, and are mainly focused on self-reported creative personality or creative tendencies (Ghanizadeh and Jahedizadeh 2016; Ghonsooly and Raeesi 2012; Mahmoodi-Shahrebabaki 2015). Most of these studies showed that people who were experiencing burnout judged themselves to be less creative (Ghanizadeh and Jahedizadeh 2016; Ghonsooly and Raeesi 2012; Mahmoodi-Shahrebabaki 2015; Derakhshanrad et al. 2019; Drafahl 2020; Li et al. 2019).

Various cognitive components, especially executive functions, such as flexibility, working memory, cognitive control, and inhibition control, are fundamental to creative thinking (Benedek and Fink 2019). At the same time, previous studies showed a strong correlation between burnout and impairments in these cognitive functions. Yet, it remains unclear whether these cognitive impairments reflect specific deficits in executive subdomains, such as task switching, inhibition, or updating, or whether they are better characterized as a more generalized cognitive deficit (Deligkaris et al. 2014). For that reason, impairments in general cognitive functions may play a mediating role between burnout and subjectively reported deficits in creativity (Derakhshanrad et al. 2019; Drafahl 2020; Ghanizadeh and Jahedizadeh 2016; Mahmoodi-Shahrebabaki 2015; Li et al. 2019), as well as objectively measured deficits in psychometric creativity tasks, such as the Torrance Tests of Creative Thinking (TTCT; Torrance 1966).

Furthermore, in previous research, only non-clinical samples of the working population were included. Accordingly, more severe burnout cases were underrepresented in these samples. Throughout the course of history, creativity has been linked to many different forms of mental disorders, and there appears to be some consensus that at least less severe manifestations of psychopathology are associated with enhanced creativity (for more information on this issue, please see e.g., Fink et al. 2012; Leutgeb et al. 2016). Moreover, Oosterholt et al. (2014) showed that clinical burnout patients and non-clinical burnout individuals reported more severe cognitive problems than healthy individuals, but impairment in objective cognitive tests was found in clinical burnout patients only. In the current paper, a clinical approach was used to conceptualize burnout, as suggested by Van Dam (2021). The term clinical burnout was used for patients with a clinical burnout diagnosis, treated in a specialized psychosomatic clinic, in contrast to non-clinical samples from the working population, who reported symptoms of burnout, but were not diagnosed by a psychiatrist or clinical psychologist (Oosterholt et al. 2014; Van Dam 2016). Therefore, the aim of the present study was to examine differences in various facets of creativity between three groups with different severities of burnout: clinical burnout patients and two groups from a non-clinical working population showing high vs. low burnout symptoms. As self-assessments may be heavily distorted in people suffering from burnout, this study also included psychometric measures of figural and verbal creativity. These psychometric measures of creativity are considered useful estimates of creative potential (cf. Runco and Acar 2012) and assess both more quantitative measures of creativity, such as ideational fluency, and more qualitative aspects, such as originality.

Furthermore, we focused on several factors that might influence creativity in burnout patients, such as depression, sleep quality, daytime fatigue, and cognitive measures (i.e., selective attention and executive control).

## 2. Materials and Methods

### 2.1. Participants

In total, creativity data were analyzed in a sample of n = 176, which covered all types of occupational groups. From a previous project (Bauernhofer et al. 2018; Canazei et al. 2018), a subgroup of hospitalized patients with a clinical burnout diagnosis and high burnout scores (n = 75; hereinafter referred to as “clinical burnout” group) who performed the complete creativity test battery was included. Due to the severity of their illness, these participants were enrolled in a five-week rehabilitation program in two specialized clinics in Austria, and all patients were on sick leave for at least one week before admission. All patients were diagnosed using a semi-structured interview by a team of psychiatrists and clinical psychologists at the time the study was conducted (between 2011–2017), the ICD11 was not released; therefore, the diagnosis was based on the ICD-10 criteria of work-related neurasthenia (World Health Organization 1994), which has been suggested as the psychiatric equivalent of clinical burnout (Schaufeli et al. 2001; Van Dam 2021). Furthermore, all patients were screened for a past diagnosis of major depression using the Structured Clinical Interview for Axis I DSM-IV Disorders (SCID-I; First et al. 1997). Corresponding to former studies (Oosterholt et al. 2014; Van Dam 2016), Maslach Burnout Inventory (MBI) cut-off scores were additionally applied as a categorization variable to substantiate the diagnosis of burnout. To be included in the present study, patients needed to present an MBI exhaustion score greater than or equal to 3.5, reflecting the presence of severe burnout symptoms (Kleijwen et al. 2013).

Additionally, 101 working individuals from Austria were recruited via local advertisements or social networking, and were assigned to (1) a group of healthy participants (n = 62; hereinafter referred to as “healthy” group) with low burnout (MBI exhaustion score < 3.5) and depression scores (BDI < 10) or (2) a group of working people (n = 39; hereinafter referred to as “non-clinical burnout” group) with strongly elevated scores in the core burnout measure for emotional exhaustion (MBI exhaustion score ≥ 3.5).

The study was conducted in accordance with the 1964 Declaration of Helsinki and was approved by the authorized ethics committee (ethical code number: not included, because of anonymous peer review; approval date: not included, because of anonymous peer review). After being informed in detail about the purpose of the study, all participants provided written informed consent.

### 2.2. Measurements

#### 2.2.1. Psychometric Measures

**Beck Depression Inventory II (BDI; Hautzinger et al. 2009):**

On the BDI, participants self-report a variety of current depressive symptoms, which are rated on a scale from 0 (absent or mild) to 3 (severe) for 21 items in total. The total BDI score ranges from 0 to 63 and is computed by summing up all item scores. Hautzinger et al. (2009) reported Cronbach’s alphas up to .92, along with significant and high correlations (in a range between .68 and .89 for different samples) with similar measures.

**Maslach Burnout Inventory (MBI-GS-D; German version for general professions (Schaufeli et al. 1996; German version: Büssing and Glaser 1998)):**

The MBI-GS-D measures burnout symptoms with 16 items, scored on a 6-point Likert scale (1 = “never”, 6 = “very often”), which are classified into three dimensions. The emotional exhaustion scale and the cynicism/depersonalization scale each consists of five negatively worded items (emotional exhaustion scale: e.g., “I feel burned out from my work”; cynicism/depersonalization scale: e.g., “I have become less interested in my work since I started this job”). The personal accomplishment scale includes six positively worded items (e.g., “I feel confident that I am effective at getting things done”). As all participants in the clinical burnout group were on sick leave during the test administration, they were instructed to fill in the items of the MBI-GS-D according to how they would feel if they were working at that moment.

**Pittsburgh Sleep Quality Index (PSQI; Buysse et al. 1989):**

The PSQI measures subjective sleep quality and sleep disturbances, which participants rate retrospectively for the last 4 weeks. The 19 PSQI items produce the following seven component scores: sleep latency, sleep duration, sleep quality, sleep efficiency, sleep disturbances, use of sleeping medication, and daytime dysfunction (all ranging from 0 to 3). The global PSQI score is calculated by summing up the scores of the seven factors, and thus ranges from 0 to 21, with lower scores indicating higher sleep quality. The test-retest reliability of the global PSQI score is .84 (Buysse et al. 1989).

**The Stanford Sleepiness Scale (SSS; Hoddes et al. 1972):**

The SSS assesses participants’ current level of sleepiness. Items are rated on a 7-point scale ranging from 1 (“feeling active and vital; alert; wide awake”) to 7 (“almost in reverie; sleep onset soon; lost struggle to remain awake”).

#### 2.2.2. Measures of General Cognitive Functions

**D2 Test (Brickenkamp 2002)**

The d2 Test measures mental concentration and selective visual attention. In the paper and pencil version, participants are instructed to cross out the letter d with two dashes out of 14 lines of letters. D’s with one, three, or four marks and p’s with any number of marks serve as distractor items. The total measure of concentration performance is quantified by the number of correctly crossed out letters. Estimates of reliability (Cronbach’s alpha) range up to .98 (Brickenkamp 2002).

**Test for the assessment of executive control and concentration (TEMEKKO: Schmid 2010)**

The TEMEKKO measures executive control and consists of a verbal, numerical, and figural subtest, lasting 14 min in total. Each subtest consists of two parts, featuring 190 items per subtest. Each item is comprised of a box with three concepts, numbers, or bars. Participants are required to cross out numbers, concepts, and bars that are ascending or descending (e.g., 5 2 1, “louse”, “chair”, “house”). To distract participants while they figured out ascending and descending concepts, numbers, and bars, the prompts randomly varied in distance, size, layout (bold face or not), and position (superscript or not). The test scores were the sums of correctly crossed out boxes with ascending or descending concepts, numbers, or bars. Reliabilities (parallel test) ranged between .79 and .96 for the different test variants and samples (Schmid 2010).

#### 2.2.3. Creativity Measures

**Creative Personality Scale (CPS; Gough 1979)**

The CPS consists of 30 adjectives, of which 18 adjectives have positive associations (e.g., confident) and 12 adjectives have negative associations (e.g., conservative) with creativity. Participants rate their self-perceived personality and check all the adjectives that apply to them. Gough (1979) reported alpha coefficients ranging between .73 and .81 for different subgroups.

**Runco’s Ideational Behavior Scale (RIBS; Runco et al. 2001).** The German version of Runco’s Ideational Behavior Scale (RIBS) consists of 17 statements focused on one’s own ideational behavior (e.g., “I am good at combining ideas in ways that others have not tried”). Participants respond to all statements on a 6-point rating scale ranging from “not at all” (1) to “very much” (6). The total ideational behavior score is determined by adding up the individual ratings. For the original version of the scale, Runco et al. (2001) reported a Cronbach’s alpha of .92.

**Figural creativity: Torrance Tests of Creative Thinking (TTCT; Torrance 1966)**

As a measure of figural creativity, we used the first three figures of the subtest picture completion of the Torrance Tests of Creative Thinking (TTCT; Torrance 1966), for which a total time limit of 3 min was set. In this test, participants are presented with abstract lines or figures that have to be completed or extended in an original way. As an additional requirement, participants have to add original titles to their generated drawings. The following instructions (in German) were used for the Torrance Test: “By adding lines to the incomplete figures on this page and the next page, you can sketch some interesting objects or pictures. Think of pictures or objects that nobody else would think of. Find an interesting title for each drawing and write it in the line provided below the figure”.

For scoring, two raters rated the originality of each of the generated drawings on a 4-point rating scale ranging from 1 (“not original at all”) to 4 (“highly original”). Subsequently, the ratings were averaged over the items and the two raters, resulting in one average figural originality score for each participant. The inter-rater agreement was satisfactory (ICC = .835).

**Verbal creativity (Edl et al. 2014; Leutgeb et al. 2016)**

Two verbal idea generation tasks were administered, in which participants were instructed to (a) respond creatively to a given hypothetical situation (“A sound breaks the silence”), and (b) come up with creative explanations for an alteration in two pictorially presented situations (a chair with four legs vs. the same chair with only three legs). Both tasks resemble items used in common psychometric creativity tests, such as the verbal imagination subscales of the Berlin intelligence test (Jäger et al. 1997) or the TTCT (Torrance 1966). For Part A of the verbal idea generation task (hypothetical situation: e.g., “A sound breaks the silence”), the following instructions were used (in German): “In the following, unusual hypothetical situations are presented to you. Please find as many different explanations as possible. Please also think of things that nobody else would think of. Write down your ideas in keywords as quickly as possible”. Afterward, a practice example with 4 possible explanations for the unusual hypothetical situation was presented.

The following instructions were used for Part B of the verbal idea generation task (creative explanations for an alteration in two pictorially presented situations, e.g., a chair with four legs vs. the same chair with only three legs): “Please look at the given pictures carefully and explain how the change from the initial state (left picture) to the final state (right picture) may have occurred. Find as many different explanations as possible. Do not forget about unusual things. But keep in mind that the answers should not be impossible! There are no right or wrong answers”. Again, a practice example with 4 possible explanations for an alteration in two pictorially presented situations was presented.

Testing time was restricted to three minutes for each task. Performance in the verbal idea generation tasks was scored for ideational fluency (number of generated non-identical ideas), as well as originality. For the latter, two raters provided an overall rating (i.e., “snapshot” scoring; Silvia et al. 2009) for the originality of the generated ideas for each item and participant on a 4-point rating scale ranging from 1 (“not original at all”) to 4 (“highly original”). Then, the ratings were averaged over items and raters, resulting in one average verbal originality score for each participant. The inter-rater agreement was again satisfactory (hypothetical situation: ICC = .84; change of situation: ICC = .79).

### 2.3. Statistical Analysis

All statistical procedures were calculated in SPSS 26. Demographic characteristics of the three study groups were compared by means of analyses of variance (ANOVAs), Pearson’s chi-square tests (gender, education), and Fisher’s exact test (psychotropic medication; Table 1).

Group differences in the psychometric variables were tested by means of multivariate and univariate analyses of variance. This set of analyses allowed us to address the differences in specific clinical and individual characteristics of hospitalized burnout patients from non-clinical burnout patients and healthy controls. Importantly, this analysis approach allowed us to detect possible non-linear associations (e.g., creativity in clinical/psychiatric samples sometimes following an inverted U-shaped function, indicating that less severe manifestations of clinical conditions were associated with elevated levels of creativity, cf. Abraham 2014; Fink et al. 2014). As in some cases (BDI, PSQI, and SSS), the assumption of homogeneity of variance in the ANOVAs was violated, and one-way Welch ANOVAs were run for control purposes. These analyses yielded the same pattern of results as obtained by the ANOVAs. Tukey’s post-hoc tests were used to test specific differences between the three study groups.

In addition, this study explored the manifold associations (Pearson correlations) between the different facets of creativity with clinical characteristics, such as depression, sleep quality, daytime-fatigue, and cognitive measures (cognitive control and concentration ability). This set of analyses would provide a first look into which variables were most critical or sensitive to the measured facets of creativity in participants, covering a broad range of burnout manifestations (e.g., is creativity more strongly associated with affective or cognitive factors?). Based on these initial findings, we expected to gain a better understanding of which factors could support or impede creativity in burnout, revealing exciting new hypotheses for future research.

All statistical analyses were run with a significance level of α = .05. Effect size indices were given in terms of eta-squared (*η*^2^).

## 3. Results

### 3.1. Sociodemographic Data

Sociodemographic data are presented separately for the three study groups in Table 1.

The three groups differed in age, *F*(2, 173) = 8.55, *p* < .001 and average working-time *F*(2, 173) = 5.16, *p =* .007, but recall bias may have occurred, as the clinical burnout group, on average, had been on sick leave for four and a half months. The three groups did not differ in terms of sex, level of education, and various work-situation variables (all *p_s_* ≥ .1), but frequency of psychotropic medication taken was higher in the clinical burnout group (71%) compared with the nonclinical burnout group and healthy groups (5% and 0%, respectively; all *p_s_* < .001; Fisher’s exact test).

### 3.2. Clinical Characteristics and Cognitive Performance

Clinical characteristics and cognitive performance are presented in Table 2.

#### 3.2.1. Depression and Burnout Symptoms

**Beck Depression Inventory (BDI):** The three groups differed in their BDI scores, *F*(2, 173) = 152.32, *p* < .001, *η*^2^ = .64. The mean depression score was highest in the clinical burnout group, followed by the non-clinical burnout group, and finally, by the healthy group (all *p_s_* < .001).

**Maslach Burnout Inventory (MBI-GS-D):** Additionally, significant differences were found in all three MBI scales: emotional exhaustion (*F*(2, 173) = 284.65, *p* < .001, *η*^2^ = .77), cynicism/depersonalization (*F*(2, 173) = 88.56, *p* < .001, *η*^2^ = .51), and personal accomplishment (Welch’s *F*(2, 173) = 17.34, *p* < .001, *η*^2^ = .17). Post-hoc tests showed significant differences between all three groups in the two MBI-GS scales, emotional exhaustion and cynicism/depersonalization (all *p_s_* < .001). The clinical burnout group showed the highest scores and the healthy group had the lowest scores in emotional exhaustion and cynicism/depersonalization (see Table 3). In the MBI-GS personal accomplishment scale, the clinical burnout group showed lower scores compared with the non-clinical burnout group and the healthy group (all *p_s_* < .004). No difference was found between the non-clinical burnout and healthy group for this scale (*p =* .205).

#### 3.2.2. Self-Rated Sleepiness

**Daytime sleepiness—Stanford Sleepiness Scale (SSS):** Self-rated daytime sleepiness (SSS) was different between the three groups, *F*(2, 173) = 28.36, *p* < .001, *η*^2^ = .25. Post-hoc tests showed significantly higher scores for daytime fatigue and sleepiness in the clinical burnout group compared with the healthy group (*p* < .001), as well as with the non-clinical burnout group (*p* = .001). In contrast, SSS scores did not differ between the non-clinical burnout and healthy groups (*p* = .143).

**Nighttime sleep quality—Pittsburgh Sleep Quality Index (PSQI):** We observed different PSQI scores between the three groups, *F*(2, 173) = 67.98, *p* < .001, *η*^2^ = .44. Post-hoc tests revealed significantly lower sleep quality (i.e., higher scores on the PSQI) in the clinical burnout group compared with the non-clinical burnout group, as well as with the healthy group (all *p*_s_ < .001). Furthermore, the non-clinical burnout group had higher PSQI scores compared with the healthy group (*p* = .018).

#### 3.2.3. Cognitive Measures

**Sustained attention (d2):** The d2 concentration score was different between the three groups, *F*(2, 173) = 23.08, *p* < .001, *η*^2^ = .21. Post-hoc tests showed lower concentration scores in the clinical burnout group compared with the non-clinical burnout group and the healthy group (*p*_s_ < .001). The d2 concentration scores did not differ between the non-clinical burnout and healthy groups (*p* = .843).

**Executive control and concentration performance (TEMEKKO):** We observed different attentional performance scores (TEMEKKO, overall score) between the three groups, *F*(2, 173) = 5.01, *p* = .008, *η*^2^ = .06. Post-hoc analysis revealed significantly lower concentration scores in the clinical burnout group compared with the non-clinical burnout group (*p* = .008). The other single comparisons were not significant (*p_s_* > .1; see Table 3).

#### 3.2.4. Creativity Measures

Self-assessed creative ideation and creative personality, as well as psychometric measures of figural and verbal creativity, are presented in Table 3.

**Table 3:** Self-assessed creative ideation and creative personality, as well as psychometric measures of figural and verbal creativity.

	Clinical Burnout Group	Non-Clinical Burnout Group	Healthy Group
	n = 75	n = 39	n = 62
**Self-assessed creative ideation and creative personality**			
Creative personality scale (CPS)	1.55 (4.48)	5.82 (3.11)	7.23 (3.63)
Runco’s ideational behavior scale (RIBS)	52.81 (18.91)	64.10 (16.80)	61.27 (19.55)
**Psychometric measures of figural and verbal creativity**			
Figural creativity (originality)	2.02 (0.66)	2.34 (0.65)	2.35 (0.64)
Verbal creativity (fluency)	7.11 (3.0)	7.63 (2.48)	7.54 (2.79)
Verbal creativity (originality)	2.05 (0.78)	2.45 (0.84)	2.15 (0.76)

**Note:** The table shows means (standard deviations). The originality scores range between 1 (“not original at all”) to 4 (“highly original”).

A multivariate analysis of variance (MANOVA) based on the five creativity measures revealed a significant multivariate main effect group (*F* (10,340) = 8.52, *p* < .001, *η*^2^ = .20). Subsequent univariate analyses indicated significant effects in all creativity measures (CPS: *F* (2,173) = 38.48, *p* < .001, *η*^2^ = .31; RIBS: *F* (2,173) = 5.89, *p* = .003, *η*^2^ = .06; figural creativity: *F* (2,173) = 5.48, *p* = .005, *η*^2^ = .06; verbal creativity/originality: *F* (2,173) = 3.31, *p* = .039, *η*^2^ = .04), except for ideational fluency in the verbal creativity task (*F* (2,173) = 0.60, *p* = .550, *η*^2^ = .01).

The pattern of group differences was strikingly similar across the creativity measures. The clinical burnout group scored lowest in all creativity measures (see Table 3). In accordance with this, all post hoc tests revealed significant (*p* < .05) differences between the clinical burnout group and both other groups, except for Verbal Originality, where no differences between the clinical burnout group and healthy group were found. The non-clinical burnout and healthy groups did not differ in creativity.

Correlations between the creativity measures and sleep variables, executive control/general cognitive ability, depression, and MBI burnout dimensions in the total sample of participants are shown in Table 4. All creative performance variables (TTCT Originality, Fluency, and Originality in the verbal creative ideation tasks) and both self-assessed creativity measures (CPS and RIBS) were significantly and positively correlated with executive control and sustained attention (all *p_s_* < .05), whereas there were no substantial correlations of creative task performance with the Maslach dimensions, BDI score, or sleep parameters (apart from the correlations of figural originality with MBI emotional exhaustion, BDI, and the Stanford Sleepiness Scale). The MBI dimensions, as well as the sleep parameters and BDI score were significantly associated with self-assessed ideational fluency and the creative personality scale (all *p_s_* < .05). 

## 4. Discussion

The present study aimed at specifying the relations of burnout symptoms with self-assessed measures of creative ideation, creative personality, and psychometric measures of figural and verbal creativity, which are known as useful estimates of creative potential (Runco and Acar 2012). Studying three groups with strongly varying burnout symptoms allowed us to examine the importance of burnout symptom severity in these relationships, which is novel in the literature.

The clinical burnout group scored lowest on all cognitive tests, including executive control and concentration ability, and in nighttime sleep quality and depression. Though some studies have revealed cognitive impairments in people with burnout symptoms (Grossi et al. 2015; van Dijk et al. 2020; for overview of studies, see Riedrich et al. 2017), very little is known about the relationship between creativity and burnout, especially in samples presenting more severe manifestations of burnout. This study provides a clear answer to this question: more severe (i.e., clinical) manifestations of burnout are linked to lower levels of creativity, including self-assessed facets of creativity (creative personality and ideational fluency) and psychometrically determined creative task performance, both in the verbal and figural creativity domains. These findings do not seem surprising in the face of the overwhelming body of evidence linking creativity to various “bright” sides of personality, such as motivation (Collins and Amabile 1999), positive affect (Ashby et al. 1999; Baas et al. 2008), and openness to experience (Feist 1998). Creativity has even been considered a sign of mental health and emotional well-being (Simonton 2000). The core characteristics of burnout, on the other hand, involve emotional exhaustion, depersonalization, and reduced personal accomplishment (cf. Maslach and Jackson 1981), sharing common characteristics with other clinical conditions, such as chronic fatigue or depression. However, higher levels of creativity have sometimes also been linked to more “darker” facets of personality, especially to personality traits such as psychoticism (Acar and Runco 2012), disorders on the schizophrenia spectrum (Acar et al. 2018), and bipolar disorder (Andreasen 2008). All of these conditions appear to be of minor relevance to the context of burnout. However, the current understanding of the relationship between creativity and psychopathology is that these two constructs are related in an inverted U-shaped manner, suggesting that more severe manifestations of psychopathology impede creativity to a greater extent (Abraham 2014). In line with this concept, our results revealed that the non-clinical burnout group mostly showed comparable creativity scores to the healthy group, indicating that only more severe (i.e., clinical) manifestations of burnout were detrimental to creativity.

This study also provides a preliminary answer to the question of the most critical factors involved in different facets of creativity in a sample that covers a broad range of burnout manifestations. As shown in Table 4, both executive cognitive control and sustained attention were significantly and positively correlated with all creative task performance measures and self-assessed facets of creativity (CPS and RIBS). The clinical variables (sleep variables, MBI dimensions, and depression score) were strongly linked to both self-assessed facets of creativity, but were only marginally linked to psychometrically determined creative task performance. This finding is interesting, as it suggests that cognitive functions, rather than affective variables (such as depression or emotional exhaustion), are mainly responsible for impaired creative task performance in burnout—a construct whose core definitions primarily involve affective functions. Depressive symptoms seem to only play a role when participants are required to assess the presence/absence of personality characteristics that are either supportive or detrimental to creativity (CPS). These findings were consistent with the current creativity literature, showing that cognitive functions are important driving forces for creativity (see e.g., Benedek et al. 2014; Benedek and Fink 2019; Zabelina and Robinson 2010).

The present study has some limitations. First, due to the correlational/cross-sectional design of this study, causality and direction of influence cannot be assessed. Second, in the current paper, we used similar inclusion criteria for the clinical burnout and non-clinical burnout groups as previous investigations (e.g., the diagnosis for clinical burnout was based on the ICD-10 criteria of work-related neurasthenia; a cut-off score of ≥3.5 on the exhaustion scale in the MBI-GS_D was used for the clinical and non-clinical burnout groups (see Oosterholt et al. 2014; Van Dam 2016). As there is no consensual definition of burnout, the comparability and generalizability of our results may be limited. Furthermore, in the present study, we cannot exclude a recall bias, especially in the clinical burnout patients who reported longer sick leaves. Moreover, we cannot exclude the potential effects that comorbid disorders had on creativity measures, as we did not administer the Structured Clinical Interview for Axis I DSM-IV Disorders (SCID-I; First et al. 1997) or the Mini-International Neuropsychiatric Interview (MINI; Sheehan et al. 1998). A further limitation of the current study is that we do not have any information about how the healthy controls and non-clinical high burnout group compared with the clinical burnout patients on their prior cognitive abilities (before diagnosis). Furthermore, at the time of the investigation, 71% of the clinical burnout patients received psychotropic medication, particularly antidepressant drug therapy. Thus, we cannot exclude possible medication effects on the performance of creativity and cognitive tests. However, it should be noted that in the research of the influence of antidepressant drug therapy on cognitive function, the direction of effects is quite controversial (Orzechowska et al. 2015). Finally, it should be mentioned that although the group of clinical burnout patients was slightly older than the other groups, this should not strongly impact our pattern of findings, as younger and older people have been shown to be similarly capable of divergent thinking (for a systematic review, see Fusi et al. 2020).

Taken together, our results showed that more severe (i.e., clinical) manifestations of burnout were linked to impaired creativity, including self-assessed facets of creativity (creative personality and ideational fluency) and psychometrically determined creative task performance, both in the verbal and figural domains. In line with the concept that creativity and psychopathology are related in an inverted U function, our results revealed that creativity in the non-clinical burnout and healthy groups was mostly comparable. Furthermore, poorer cognitive functions were related to lower creative task performance, but only a few clinical variables were related to lower creative task performance, suggesting that cognitive functions, rather than affective variables, are primary facilitators of creativity in burnout patients. A practical implication of the current study is that improving general cognitive functions, such as executive cognitive control, may be a promising way to enhance creative modes of thinking in burnout patients, which in turn may be a promising avenue in the prevention and rehabilitation of burnout. Therefore, treatment programs for burnout should consider adding cognitive tests to the diagnosis of burnout, as well as cognitive training methods that would enhance executive control.

## Figures and Tables

**Table 1:** Sociodemographic characteristics.

	Clinical Burnout Group	Non-Clinical Burnout Group	Healthy Group
	(n = 75)	(n = 39)	(n = 62)
**Age (years)**	44.94 (7.91)	37.69 (10.74)	39.68 (11.3)
**Sex:**			
Female (%)	67%	54%	66%
Male (%)	33%	46%	34%
**Sick-leave days**	140.72 (107.0)	-	-
**Level of education**			
1: Less than high school	47%	41%	44%
2. High school graduate	37%	23%	32%
3. Some college	16%	36%	24%
**Work-situation variables**			
Average working hours/week	41.53 (13.74)	34.64 (16.89)	34.44 (13.26)
Working in night shifts	11%	8%	15%
Leadership position	37%	38%	31%
**Contract**			
Full-time employee	60%	46%	52%
Part-time employee	32%	41%	29%
Self-employed	8%	13%	19%
**Psychotropic medication**	71%	5%	0%

Note: The table shows means (standard deviations). Level of education was measured in terms of highest level of education completed, ranging from 1 (less than high school) to 4 (some college).

**Table 2:** Clinical characteristics and cognitive performance.

	Clinical Burnout Group	Non-Clinical Burnout Group	Healthy Group
	n = 75	n = 39	n = 62
**Depression and burnout symptoms**			
**Beck Depression Inventory**	23.04 (9.24)	8.29 (6.98)	2.57 (2.64)
**Maslach Burnout Inventory**			
Emotional exhaustion	5.23 (0.68)	4.13 (0.57)	2.56 (0.66)
Cynicism/Depersonalization	4.49 (0.80)	3.42 (1.15)	2.23 (1.07)
Personal accomplishment	4.43 (0.75)	4.91 (0.57)	5.17 (0.82)
**Scales for daytime fatigue and nighttime sleep quality**			
Stanford Sleepiness Scale (SSS)	3.44 (1.24)	2.49 (1.09)	2.06 (0.87)
Sleep quality (PSQI):	10.20 (4.14)	5.59 (2.89)	3.73 (2.34)
**Cognitive measures**			
Concentration performance:(d2 test)	140.01 (34.01)	189.1 (53.06)	184.03 (49.71)
Executive performance:(TEMEKKO)	36.12 (8.55)	41.39 (7.95)	39.23 (9.65)

Note: The table show means (standard deviations). PSQI: Pittsburgh Sleep Quality Index; TEMEKKO: Test for the assessment of executive control and concentration.

**Table 4:** Pearson’s correlations between creativity and clinical characteristics, as well as cognitive performance, in the total sample of participants (n = 176).

	CPS	RIBS	Figural Creativity (Originality)	Verbal Creativity (Fluency)	Verbal Creativity (Originality)	BDI	MBI (EE)	MBI (ZY)	MBI (PA)	SS	PSQI	TEMEKKO	d2
CPS	1	.570 **	.114	.204 **	.267 **	−.624 **	−.539 **	−.496 **	.425 **	−.432 **	−.490 **	.211 **	.369 **
RIBS		1	.058	.206 **	.270 **	−.274 **	−.213 **	−.194 **	.201 **	−.231 **	−.081	.253 **	.177 *
Figural Creativity (Originality)			1	.081	.313 **	−.189 *	−.194 **	−.074	.027	−.149 *	−.140	.247 **	.263 **
Verbal Creativity (Fluency)				1	.414 **	−.023	−.051	−.041	−.001	−.115	−.037	.243 **	.166 *
Verbal Creativity (Originality)					1	−.070	−.024	.006	−.009	−.097	−.073	.356 **	.275 **
BDI						1	.728 **	.658 **	−.442 **	.519 **	.719 **	−.199 **	−.380 **
MBI (EE)							1	.750 **	−.432 **	.523 **	.602 **	−.182 *	−.316 **
MBI (CY)								1	−.613 **	.456 **	.475 **	−.164 *	−.253 **
MBI (PA)									1	−.313 **	−.313 **	.088	.159 *
SS										1	.426 **	−.087	−.262 **
PSQI											1	−.069	−.285 **
TEMEKKO												1	.600 **
d2													1

**Note:** * *p* < .05, ** *p* < .01; CPS = Creative personality scale; RIBS = Runco’s ideational behavior scale; TTCT (Originality) = Torrance Tests of Creative Thinking: Originality score; BDI = Beck Depression Inventory; MBI (EE) = Maslach Burnout Inventory: Emotional exhaustion subscale; MBI (CY) = Maslach Burnout Inventory: Cynicism/depersonalization subscale; MBI (PA) = Maslach Burnout Inventory: Personal accomplishment subscale; SS = Stanford Sleepiness Scale; PSQI: Pittsburgh Sleep Quality Index; d2 = d2 concentration score; TEMEKKO: Test for the assessment of executive control and concentration.

## Data Availability

The datasets generated and/or analyzed during the current study are available from the corresponding author upon reasonable request.
